# Functional plasticity of the ipsilateral primary sensorimotor cortex in an elite long jumper with below-knee amputation

**DOI:** 10.1016/j.nicl.2019.101847

**Published:** 2019-05-09

**Authors:** Nobuaki Mizuguchi, Kento Nakagawa, Yutaka Tazawa, Kazuyuki Kanosue, Kimitaka Nakazawa

**Affiliations:** a Faculty of Sport Sciences, Waseda University, 2-579-15 Mikajima, Tokorozawa, Saitama 359-1192, Japan; b Faculty of Science and Technology, Keio University, 3-14-1 Hiyoshi, Kohoku-ku, Yokohama city, Kanagawa 223-8522, Japan; c The Japan Society for the Promotion of Science, 5-3-1 Kojimachi, Chiyoda-ku, Tokyo 102-0083, Japan; d Graduate School of Arts and Sciences, The University of Tokyo, 3-8-1 Komaba, Meguro-ku, Tokyo 153-8902, Japan

**Keywords:** Athlete, Amputee, Motor learning, Rehabilitation, Plasticity, Prosthesis

## Abstract

Functional plasticity of the sensorimotor cortex occurs following motor practice, as well as after limb amputation. However, the joint effect of limb amputation and intensive, long-term motor practice on cortical plasticity remains unclear. Here, we recorded brain activity during unilateral contraction of the hip, knee, and ankle joint muscles from a long jump Paralympic gold medalist with a unilateral below-knee amputation (Amputee Long Jumper, ALJ). He used the amputated leg with a prosthesis for take-off. Under similar conditions to the ALJ, we also recorded brain activity from healthy long jumpers (HLJ) and non-athletes with a below-knee amputation. During a rhythmic isometric contraction of knee extensor muscles with the take-off/prosthetic leg, the ALJ activated not only the contralateral primary sensorimotor cortex (M1/S1), but also the ipsilateral M1/S1. In addition, this ipsilateral M1/S1 activation was significantly greater than that seen in the HLJ. However, we did not find any significant differences between the ALJ and HLJ in M1/S1 activation during knee muscle contraction in the non-take-off/intact leg, nor during hip muscle contraction on either side. Region of interest analysis revealed that the ALJ exhibited a greater difference in M1/S1 activity and activated areas ipsilateral to the movement side between the take-off/prosthetic and non-take-off/intact legs during knee muscle contraction compared with the other two groups. However, difference in activity in M1/S1 contralateral to the movement side did not differ across groups. These results suggest that a combination of below-knee amputation and intensive, prolonged long jump training using a prosthesis (i.e. fine knee joint control) induced an expansion of the functional representation of the take-off/prosthetic leg in the ipsilateral M1/S1 in a muscle-specific manner. These results provide novel insights into the potential for substantial cortical plasticity with an extensive motor rehabilitation program.

## Highlights

• A Paralympic gold medalist with a unilateral below-knee amputation was recruited.
• Brain activity during hip, knee, and ankle movements was recorded.
• Brain activity was compared with healthy athletes and non-athletes with amputation.
• Greater ipsilateral M1/S1 activity during knee movement was observed in the medalist.
• Intensive motor practice and limb amputation would induce drastic neural plasticity.

## 1 Introduction

Neural plasticity occurs with motor practice (Dayan and Cohen, 2011). A typical example involves the expansion of representation in the primary motor cortex (M1) associated with a trained limb (Karni et al., 1995; Pascual-Leone et al., 1995). In addition to functional plasticity, structural changes in M1 are also induced by motor practice (Sampaio-Baptista et al., 2013; Taubert et al., 2016). Because the neural plasticity caused by motor practice is likely induced in a non-linear manner (Dayan and Cohen, 2011; Jäncke et al., 2009), investigating brain changes in professional sports players or musicians should help to clarify the long-term effects of motor practice on cortical plasticity in humans. Indeed, brain activity and structure in elite athletes and musicians differ from what is seen in healthy individuals or mid-level players (Krings et al., 2000; Nakata et al., 2010; Naito and Hirose, 2014; Huang et al., 2015). For example, activity in M1 in a top soccer player during ankle movements was very weak and limited compared with that of other professional soccer players (i.e. neural efficiency) (Naito and Hirose, 2014).

Limb amputation also induces large-scale neural plasticity and reorganization in cortical and subcortical regions. Electrophysiological and neuroimaging studies have shown that limb amputation induces an expansion of the cortical representation of neighboring intact body parts of the amputated limb (Florence and Kaas, 1995; Striem-Amit et al., 2018). For example, arm representation took over regions that previously received input from an amputated hand (Florence and Kaas, 1995). However, recent studies have suggested that the sensory topography of amputated fingers remain even years after amputation, and that activity changes in cortical regions originate from the formation or potentiation of new connections in subcortical regions such as the brainstem (Kikkert et al., 2016; Makin and Bensmaia, 2017). Activity changes induced by amputation was also observed in the sensorimotor regions ipsilateral to the amputated limb. Indeed, limb amputation decreases the strength of interhemispheric connectivity within the sensorimotor network (Pawela et al., 2010) and alters the microstructure of the corpus callosum (Simões et al., 2012; Li et al., 2017). In addition, the degree of cortical plasticity in motor regions was reported to be associated with the daily use of a prosthesis in amputees (Karl et al., 2004). The intensive use of the intact limb in unilateral upper limb amputees has been investigated by Philip and Frey (2014). They demonstrated that activity within the former cortical sensorimotor hand territory in the left hemisphere of amputees (with amputation of the dominant right hand) was increased by intensive use of the nondominant left hand. However, cortical plasticity that occurs in association with unilateral amputation and intensive, long-term practice that involves prosthesis control by the remaining portion of the amputated limb has not been investigated.

Considering the existing evidence, limb amputation and intensive, long-term motor practice that involves the use of a prosthesis would induce drastic cortical plasticity in M1. To investigate this, we recorded brain activity during unilateral muscle contractions of the hip, knee, and ankle from a long jump Paralympic gold medalist with a unilateral below-knee amputation (Amputee Long Jumper, ALJ). We compared his brain activity with that of healthy long jumpers (HLJ), and non-athletes with a below-knee amputation (Amputee Non-Athletes, ANA). The elite long jumper with the below-knee amputation took off using the amputated leg with a prosthesis. Thus, fine control of the knee joint of the amputated leg is critical for a good long jump performance. Indeed, a recent biomechanical study demonstrated that his take-off is mechanically efficient (Willwacher et al., 2017). Thus, we hypothesized that his M1 activity during knee movement of the prosthetic leg would be different from that seen in healthy long jumpers and non-athletes with a similar amputation and prosthesis.

## 2 Materials and methods

### 2.1 Participants

One long jump Paralympic gold medalist with a unilateral below-knee amputation (Amputee Long Jumper, ALJ), twelve healthy long jumpers (HLJ), and four non-athletes with a unilateral below-knee amputation (Amputee Non-Athletes, ANA) participated in the experiment. At 14 years old, the ALJ had had his right leg amputated below the knee after an accident. Subsequently, he received long jump training, using his prosthesis for take-off. He won gold medals at the Summer Paralympics in 2012 and 2016, and holds the long jump world record in his category (T44, 8.40 m). He was 27 years old at the time of the experiment. Other general participant characteristics are shown in Table 1. All participants received a detailed explanation of the experimental procedures before the experiment and provided written informed consent. The study was approved by the Ethics Committee of the Graduate School of Arts and Sciences, The University of Tokyo (475-2). The experiment was carried out according to the principles and guidelines of the Declaration of Helsinki (1975).

**Table 1:** Participant characteristics.

Participants	Age (years)	Sex	Take-off / prosthetic side	Time of long jumper / amputee (years)	Best record of long jump (m)
ALJ	27	M	Right	13 (amputee)	8.40
				7 (long jumper)	

HLJ group					
1	22	M	Left	10	7.56
2	18	M	Left	7	7.34
3	20	M	Left	6	7.03
4	21	M	Right	10	7.07
5	23	M	Left	10	7.23
6	21	M	Left	10	7.48
7	21	M	Right	9	7.47
8	23	M	Right	11	7.82
9	21	M	Right	6	7.72
10	23	M	Left	9	7.49
11	19	M	Left	6	7.86
12	20	M	Left	12	7.61
Mean ± SD	21 ± 2			9 ± 2	7.47 ± 0.27

ANA group					
1	18	M	Left	13	
2	45	M	Right	24	
3	32	F	Right	32	
4	41	M	Left	37	
Mean ± SD	34 ± 12			27 ± 10	

### 2.2 Procedure

All participants conducted six motor tasks involving the use of two legs (right and left) and three joints (ankle, knee, and hip) in an MRI scanner. For knee and hip joints, participants performed unilateral rhythmic isometric contraction of the knee extensor and gluteus maximus muscles, respectively. Ankle movement involved simple cyclic dorsiflexion/plantarflexion. For ankle movement with the amputated leg, the ALJ and ANA were instructed to imagine a cyclic dorsiflexion/plantarflexion movement using the first-person perspective (Guillot et al., 2009). No participants reported experiencing phantom movements during motor imagery. Participants were instructed to perform all contractions at the same effort level. To control the frequency of movements, participants used a blinking yellow circle projected onto a black background as a pacing stimulus. In addition to the pacing stimulus, instructions for each specific task appeared on the screen, such as “R ankle” for right ankle contraction and “Rest” for a rest period. Before the MRI scan, each participant practiced six movements while EMG was recorded outside the MRI room.

Participants completed six fMRI scans. One scan consisted of six alternate repetitions of the tasks (six types of movements × one repetition) and rest periods. The order of the six movements was counter-balanced across scans. Both task and rest period durations were 20 s. Each scan included an 8-s dummy scan and a 20-s rest period before the first task. In total, each scan lasted 268 s.

### 2.3 fMRI data acquisition

All MRI images were acquired using a 3 T MR scanner with a 64-channel head coil (MAGNETOM Prisma, Siemens, Germany). Blood oxygenation level-dependent contrast functional images were acquired using T2*-weighted echo planar imaging free induction decay sequences with the following parameters: TR 2000 ms, TE 25 ms, FOV 192 mm × 192 mm, flip angle 90°, voxel size 3 mm × 3 mm × 3 mm, and gap 0.75 mm. In each scan, 130 volumes were acquired. The orientation of the axial slices was parallel to the AC-PC line.

### 2.4 EMG data acquisition

Electromyographic recordings were made from the soleus (Sol), ractus femoris (RF), and gluteus maximus (GM) using a wireless EMG system (Trigno Wireless System; DELSYS, Boston, MA, USA) that was placed over the muscle bellies on both the right and left sides. For the ALJ and ANA, lower leg muscles (Sol) on the amputated side were not measured. All signals were band-pass filtered (20–450 Hz) and sampled at 1000 Hz. The signals were then converted into a digital format with an A/D converter system (PowerLab System, AD Instruments, Sydney, Australia) and stored on a computer.

### 2.5 Whole brain analysis

The raw fMRI data were analyzed using the Statistical Parametric Mapping 12 (SPM12, Wellcome Department of Cognitive Neurology, London, UK) implemented in MATLAB (Mathworks, Sherborn, Massachusetts, USA) with the typical preprocessing pipeline. To correct for head movements, the raw fMRI data were realigned to the first volume. Realigned images were normalized to the standard space of the Montreal Neurological Institute brain (MNI brain, TPM.nii in SPM12) with affine registration. Smoothing was executed with an isotropic three-dimensional Gaussian filter with full-width at half-maximum of 8 mm. High-pass filters (128 s) were also applied and low frequency noise and global changes in the signals were removed.

The statistical analysis was performed on two levels. The first-level analysis was performed for each participant using a general linear model. We constructed a statistical parametric map of the t-statistic for the six simple contrasts, including (1) right ankle movement > rest, (2) left ankle movement > rest, (3) right knee movement > rest, (4) left knee movement > rest, (5) right hip movement > rest, and (6) left hip movement > rest. To minimize the effects of head motion artifacts, we included the six head motion parameters as nuisance regressors. Subject-specific contrast images of the estimated parameter were used to perform the second-level analysis (random-effect model). To test differences between activity in the ALJ and HLJ, we performed a two-sample *t*-test for each condition. Because brain activity during movements with the take-off leg might be different from that with the non-take-off leg, contrast images for participants with a left leg take-off were flipped (a left-to-right transformation on the x-axis) to produce a left−right reversed image.

### 2.6 ROI analysis

To compare the strengths in differences between M1 activity during take-off/prosthetic and non-take-off/intact leg movements for the ALJ and HLJ or ALJ and ANA, we extracted a mean *t*-value in the leg M1 ROI (14 mm × 14 mm × 14 mm). Coordinates of the common leg ROI (x = ±4, y = −24, z = 76) were decided according to previous studies (Taubert et al., 2016). For the ROI analysis, we additionally created a statistical parametric map of the t-statistic without smoothing in each participant to exclude the contamination effect of the other hemisphere. The processing without smoothing was the same as the whole brain analysis. In addition, the ROI in the x-axis only extended from the coordinates in a lateral direction to avoid extracting values from the other hemisphere. We ignored the selected voxels outside of the brain. To consider the effect of distance between the common leg ROI and an individual activity peak, we also extracted a mean *t*-value from individual ROIs of the same size as the common leg ROI. For example, the coordinate of an individual ROI for the right M1 during ankle movement was defined according to the peak *t*-value within the precentral gyrus for the left ankle movement. This ROI was used to extract a mean *t*-value of the contralateral (right) M1 during left ankle movement and that of the ipsilateral (left) M1 during right ankle movement (also see Table 2). That is, we extracted 24 *t*-values (leg and individual ROI × contralateral and ipsilateral M1 × 6 movements). To evaluate differences in M1 activity for the ipsilateral and contralateral M1 s, we simply subtracted the non-take-off/intact side value from the take-off/prosthetic side value. Then, we compared differences in the *t*-values among the ALJ vs. HLJ and ALJ vs. ANA using Crawford-Howell *t*-tests (Crawford and Howell, 1998).

**Table 2:** Participants' peak coordinates during movements.

Participants	R ankle			R knee			R hip			L ankle			L knee			L hip		
	X	Y	Z	X	Y	Z	X	Y	Z	X	Y	Z	X	Y	Z	X	Y	Z
ALJ	-4	−34	74	−4	−24	76	−6	−32	76	8	−26	78	8	−26	78	8	−24	80

HLJ group																		
1	−6	−32	72	−8	−24	70	−10	−26	68	6	−18	70	8	−16	64	6	−16	60
2	−6	−20	76	−12	−24	70	−14	−22	68	12	−28	78	12	−26	78	16	−28	72
3	−4	−24	62	−12	−28	68	−14	−28	68	12	−28	80	14	−24	78	14	−32	66
4	−6	−22	74	−10	−18	78	−2	−20	60	8	−18	72	8	−24	78	16	−28	74
5	−6	−18	76	−4	−18	78	−2	−14	64	10	−20	78	10	−20	78	6	−28	62
6	−4	−14	76	−4	−14	76	−10	−30	68	8	−22	76	10	−20	78	8	−22	80
7	−6	−30	74	−6	−28	76	−8	−28	78	6	−20	72	10	−26	78	10	−22	76
8	−4	−14	74	−6	−24	62	−2	−14	66	8	−24	76	10	−30	78	2	−14	64
9	4	−34	76	−10	−30	68	−12	−30	68	6	−28	76	8	−30	78	12	−28	66
10	−8	−32	72	−12	−30	72	−14	−28	68	10	−22	72	14	−24	78	18	−26	72
11	−10	−22	80	−10	−24	78	−12	−24	78	8	−14	74	8	−22	78	12	−20	74
12	−12	−18	78	−14	−22	78	−16	−22	78	6	−24	70	10	−28	78	4	−14	64

ANA group																		
1	−2	−26	70	−2	−26	70	−14	−22	80	4	−28	70	10	−20	76	12	−24	76
2	−6	−16	74	−6	−16	74	−4	−16	76	6	−14	58	8	−16	78	4	−16	74
3	−12	−20	70	−12	−32	72	−16	−30	70	6	−16	66	4	−18	78	8	−16	64
4	−6	−24	74	−2	−14	66	−22	−20	76	4	−22	72	6	−18	78	10	−16	78

The coordinates were defined accoring to the Montreal Neurological Institute (MNI) space.

To evaluate differences in activated areas in the ipsilateral or contralateral M1 between the tasks of take-off and non-take-off legs, we also extracted significant voxels within the right and left precentral gyrus (Functional Connectivity Toolbox, Neuroimaging Informatics Tools and Resources Clearinghouse, USA, https://www.nitrc.org/projects/conn) for each joint movement using FWE *p* < .05 at the voxel level. Then, we compared the differences in activated areas in M1 between the ALJ vs. HLJ and ALJ vs. ANA using Crawford-Howell *t*-tests.

*P*-values of the *t*-tests were adjusted with the use of a Bonferroni correction (i.e. ALJ vs. HLJ and ALJ vs. ANA). We also calculated a 95% confidence interval (CI) using a nonparametric bootstrapping procedure (1000 iterations) for each group. Data values are expressed as the mean ± standard deviation (SD). The data generated and analyzed in the current study are available from the corresponding author upon reasonable request.

### 2.7 Correlation analysis

To examine the relationship between brain activity and long jump performance in the HLJ, Spearman's rank correlation coefficients between difference indices (i.e. *t*-values and activated areas) and best long jump record were calculated.

### 2.8 Head movement analysis

Because lower limb movements can result in head movements in the scanner, this might have affected the results, even though six head motion parameters were used as nuisance regressors. Therefore, first, the degree of volume-to-volume head movements was calculated as the root mean square (RMS) value of the volume-to-volume difference in six motion parameters for each movement condition. Then, we used paired *t*-tests to compare whether the values differed between movement conditions. We also compared the values between the ALJ and HLJ/ANA using Crawford-Howell *t*-tests. Second, we compared the maximum head movements (i.e. x, y, and z, respectively) evaluated by a peak-to-peak distance in all six scans. The maximum head movements between the ALJ vs. HLJ and ALJ vs. ANA were tested using Crawford-Howell *t*-tests. *P*-values of the *t*-tests were adjusted using Bonferroni correction. We also calculated Spearman's rank correlation coefficients between difference indices (i.e. *t*-values and activated areas) and amount of head movement (i.e. volume-to-volume and maximum head movements).

### 2.9 EMG data analysis

For each task and rest condition, EMG signals were assessed as the RMS value of all recorded muscles within a 5-s window during a stable task performing phase. The calculated RMS value was normalized to the value of the resting condition. Then we compared the normalized values on the take-off/prosthesis and non-take-off/intact sides between the ALJ and HLJ or ANA using Crawford-Howell *t*-tests. The values obtained in the task and resting conditions were also compared within the HLJ. In addition, we assessed whether the values differed between the movement conditions using paired *t*-tests. P-values of the *t*-tests were adjusted using Bonferroni correction.

## 3 Results

### 3.1 Whole brain analysis

**Fig. 1:**
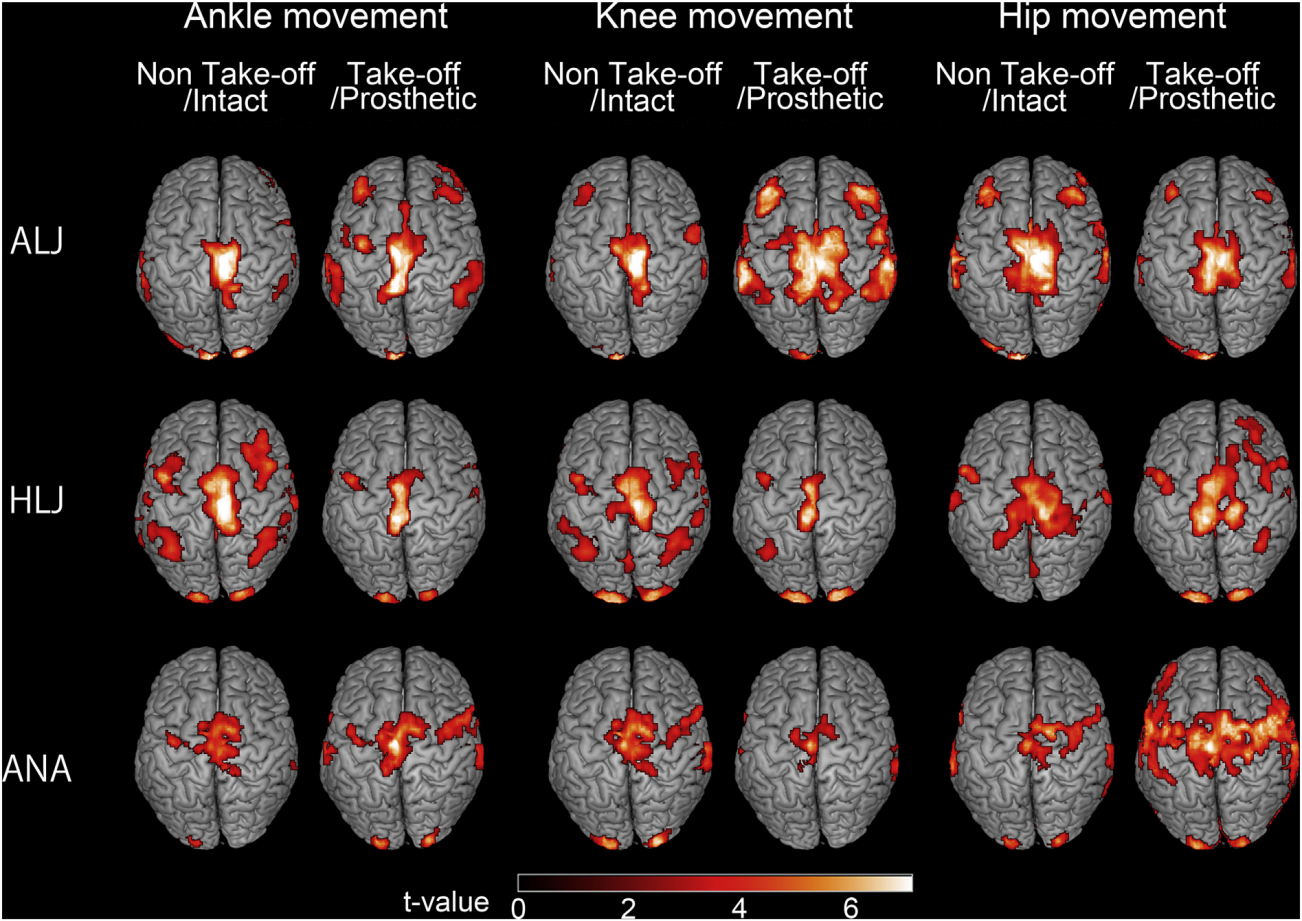
Activated regions during ankle, knee and hip movements in an elite long jumper with a below-knee amputation (ALJ), a representative healthy long jumper (HLJ: subject 7 in Table 1), and a representative non-athlete with a below-knee amputation (ANA: subject 2 in Table 1). Take-off/Prosthetic legs are displayed as right limb movements. Non-take-off/Intact legs are displayed as left limb movements. The threshold was set at a voxel level of p < .001 (uncorrected), and a cluster level of *p* < .05 (FWE).

The contralateral M1 was activated in all movement tasks for all participants. Fig. 1 shows regions that were activated during the motor tasks for the ALJ, a representative HLJ (subject 7 in Table 1), and a representative ANA (subject 2 in Table 1). Table 2 shows peak coordinates of M1 for each movement condition. No significant between-group differences were observed in x, y, or z.

**Fig. 2:**
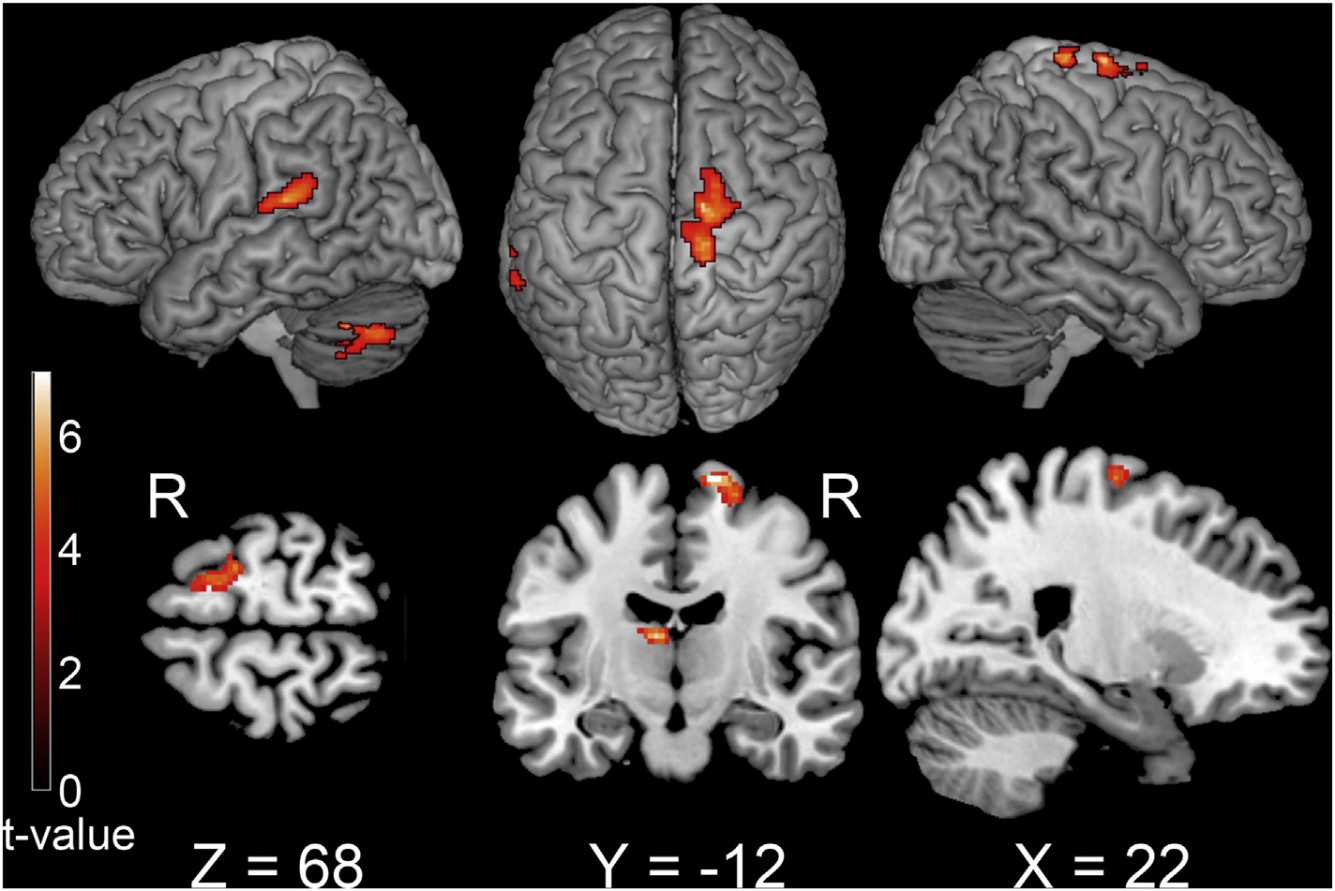
Activation differences during knee movement between an elite long jumper with a below-knee amputation and healthy long jumpers with the take-off leg. For the elite long jumper, the take-off leg had the prosthesis. All take-off legs (right and left) were adjusted to appear as the right leg. The threshold was set at a voxel level of *p* < .001 (uncorrected), and a cluster level of *p* < .05 (FWE).

Group analysis revealed that brain activity in M1 ipsilateral to the movement side during knee movement of the take-off leg for the ALJ was significantly greater than that for the HLJ (Fig. 2). In addition, the contralateral planum temporale, thalamus, and cerebellum exhibited greater activation (Table 3). However, there were no other significant differences during the other five movements, including knee movement of the non-take-off leg. These results indicated that greater activation of M1 ipsilateral to the movement side was only observed during knee movement of the take-off leg in the ALJ.

**Table 3:** Difference in activity during knee movement in the take-off leg between an elite long jumper with a lower leg prosthesis and healthy long jumpers.

Region	Side	MNI coodinates			*Z*-score	P_FWE-corr_
		X	Y	Z		cluster-level
Precentral gyrus	R	14	−12	74	4.50	0.015
Planum temporale	L	−58	−32	16	4.23	0.030
Thalamus	L	−10	−18	16	5.09	0.034
Celebellum	L	−42	−58	−34	4.45	0.047

### 3.2 Ipsilateral M1 activity using common leg ROI

**Fig. 3:**
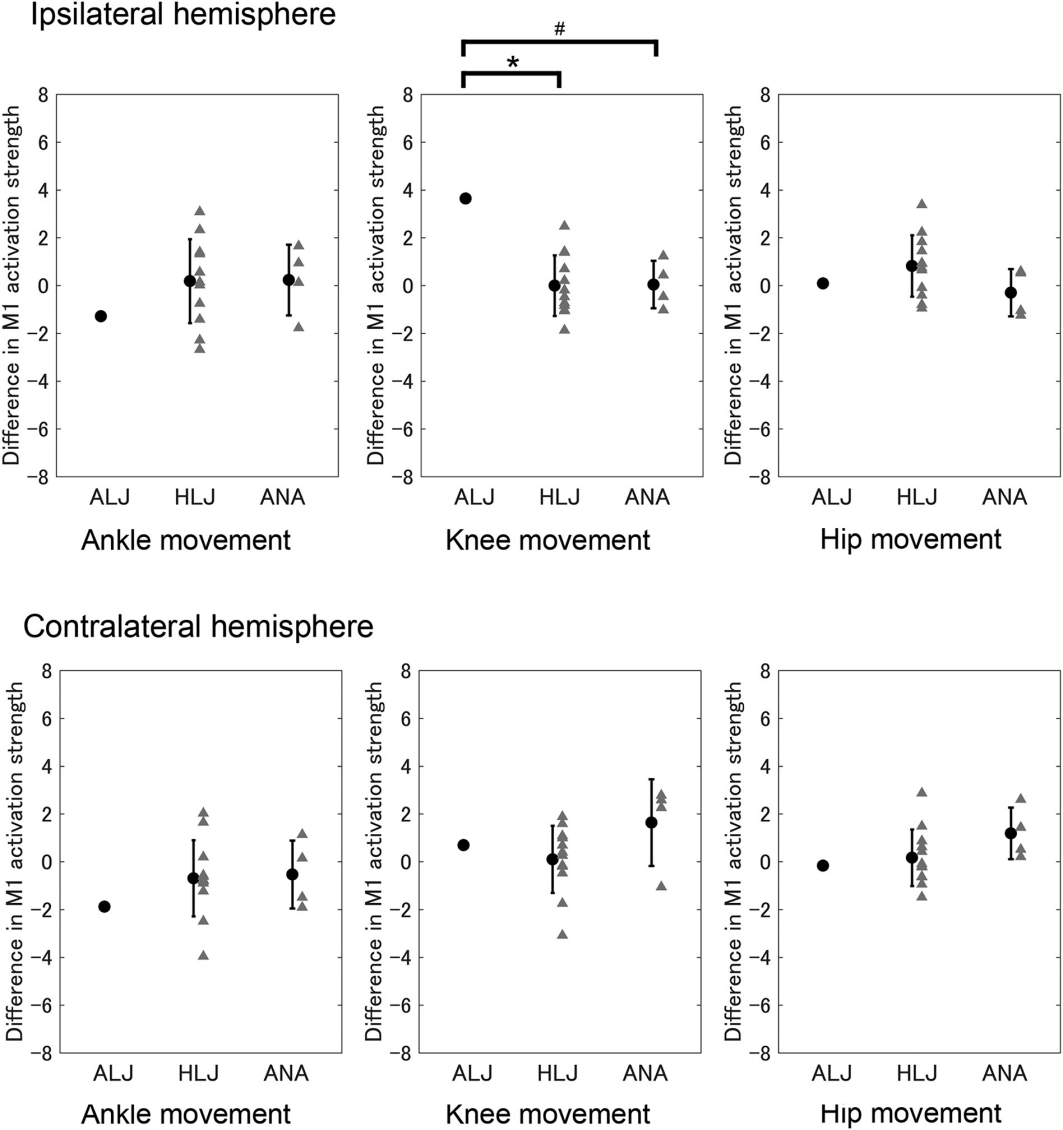
Difference in *t*-values for M1 between take-off/prosthetic and non-take-off/intact legs during knee and hip movements, respectively. Upper panels indicate M1 ipsilateral to the movement side (e.g. right hemisphere during right leg movement), and lower panels indicate M1 contralateral to the movement side. ALJ: an elite long jumper with a below-knee amputation. HLJ: healthy long jumper. ANA: non-athlete with below-knee amputation. Error bars indicate one standard deviation. *p < .05, #*p* < .10.

To evaluate the degree of difference in brain activity in M1 ipsilateral to the movement side between the take-off/prosthetic and non-take-off/intact leg movements, we calculated the difference in M1 activation strength (*t*-value) between take-off/prosthetic and non-take-off/intact leg movements. Therefore, negative values indicated that activity was greater during non-take-off/intact leg movement than take-off/prosthetic leg movement. We found that the degree of difference for the *t*-values of the ipsilateral M1 during knee movements in the ALJ (3.64) was greater than the HLJ (95% CI: −0.67–0.69) [T(11) = 2.76, *p* < .05], and tended to be greater than the ANA (95% CI: −0.45–0.53) [T(3) = 3.24, *p* = .096], (Fig. 3 upper center). This indicated that the ALJ showed greater activity in M1 ipsilateral to the movement side during knee movement using the take-off/prosthetic leg compared with the other two group. We found no significant between-group differences during ankle movements [ALJ (−1.29) vs. HLJ (95% CI: −0.75 –1.11): T(11) = 0.81, *p* = .87; ALJ vs. ANA (95% CI: −0.51–0.99): T(3) = 0.92, *p* = .85] (Fig. 3 upper left), or hip movements [ALJ (0.09) vs. HLJ (95% CI: 0.13–1.50): T(11) = 0.55, *p* = 1.00; ALJ vs. ANA (95% CI: −0.76–0.17): T(3) = 0.35, p = 1.00] (Fig. 3 upper right). These trends were similar when we performed the analysis using absolute values (i.e. irrespective of take-off/prosthetic and non-take-off/intact legs).

The ROI analysis using the leg ROI indicated that activity in M1 ipsilateral to the movement side during knee movement using the take-off/prosthetic leg was greater in the ALJ compared with the HLJ and ANA, but not during ankle and hip movements.

### 3.3 Ipsilateral M1 activity using individual ROIs

The results using individual ROIs were similar to those using the leg ROI. The degree of difference for the *t*-value of the ipsilateral M1 during knee movements was greater in the ALJ (3.55) than in both the HLJ (95% CI: −0.38–0.89) [T(11) = 2.73, *p* < .05] and the ANA (95% CI: −1.16 – -0.51) [T(3) =5.71, p < .05]. In addition, the degree of difference for the *t*-value in the ipsilateral M1 during hip movements was greater in the ALJ (2.41) than in the ANA (95% CI: −0.77–1.35) [T(3) = 5.10, p < .05] but not significantly different to that seen in the HLJ (95% CI: −0.30–1.16) [T(11) =1.47, *p* = .34]. We found no significant between-group differences during ankle movements [ALJ (−3.70) vs. HLJ (95% CI: −0.77–1.61): T(11) = 1.70, *p* = .23; ALJ vs. ANA (95% CI: −0.08–1.97): T(3) = 1.97, *p* = .29]. These trends were similar when the analysis was performed using absolute values (i.e. irrespective of take-off/prosthetic and non-take-off/intact legs).

### 3.4 Contralateral M1 activity using common leg ROI

We found no significant between-group differences during ankle movements [ALJ (−1.88) vs. HLJ (95% CI: −1.55–0.20): T(11) = 0.72, *p* = .98; ALJ vs. ANA (95% CI: −1.24–0.15): T(3) = 0.85, *p* = .92] (Fig. 3 lower left), knee movements [ALJ (0.70) vs. HLJ (95% CI: −0.65–0.84): T(11) = 0.41, *p* = 1.00; ALJ vs. ANA (95% CI: 0.77–2.54): T(3) = 0.47, p = 1.00] (Fig. 3 lower center), or hip movements [ALJ (−0.16) vs. HLJ (95% CI: −0.48–0.82): T(11) = 0.27, p = 1.00; ALJ vs. ANA (95% CI: 0.66–1.69): T(3) = 1.12, *p* = .69] (Fig. 3 lower right). These trends were similar when the analysis was performed using absolute values (i.e. irrespective of take-off/prosthetic and non-take-off/intact legs). These results indicate that the difference in activity in M1 contralateral to the movement side between take-off/prosthetic and non-take-off/intact leg movements were comparable across groups even for the knee movements.

### 3.5 Contralateral M1 activity using individual ROIs

The results using individual ROIs were similar with those using the leg ROI except for the hip movement. We found no significant between-group differences during ankle movements [ALJ (−0.14) vs. HLJ (95% CI: −0.55–0.79): T(11) = 0.20, *p* = 1.00; ALJ vs. ANA (95% CI: −1.85–0.37): T(3) = 0.23, p = 1.00], or knee movements [ALJ (0.42) vs. HLJ (95% CI: −1.25–0.35): T(11) = 0.59, p = 1.00; ALJ vs. ANA (95% CI: 1.23–3.19): T(3) = 0.77, *p* = .99]. The degree of difference for the *t*-value of the contralateral M1 during hip movements was greater in the ALJ (−4.66) than in the HLJ (95% CI: −0.73–0.48) [T(11) = 3.87, *p* < .05] and ANA (95% CI: 0.85–1.63) [T(3) = 6.64, p < .05]. Different results were obtained for the leg and individual ROI analyses when using different coordinates. These trends were similar when the analysis was performed using absolute values (i.e. irrespective of take-off/prosthetic and non-take-off/intact legs).

### 3.6 Activated area in the ipsilateral M1

**Fig. 4:**
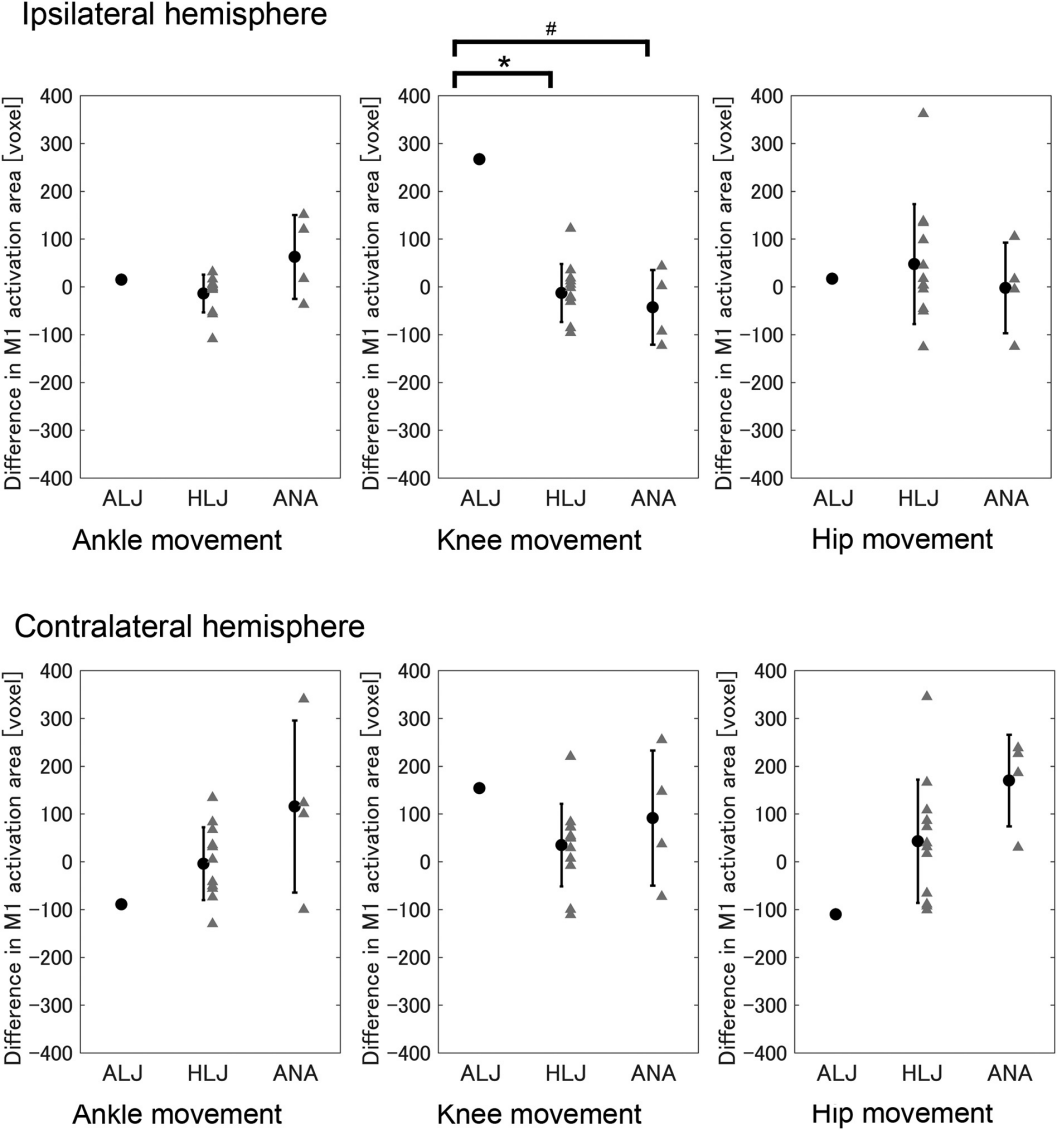
Difference in activated areas in M1 between take-off/prosthetic and non-take-off/intact legs during knee and hip movements. Upper panels indicate M1 ipsilateral to the movement side (e.g. right hemisphere during right leg movement), and lower panels indicate M1 contralateral to the movement side. ALJ: an elite long jumper with a below-knee amputation. HLJ: healthy long jumper. ANA: non-athlete with below-knee amputation. Error bars indicate one standard deviation. *p < .05, #p < .10.

The difference index for activated areas (i.e. number of activated voxels) in the ipsilateral M1 during knee movements between the take-off/prosthetic and non-take-off/intact legs in the ALJ (267) was greater than in the HLJ (95% CI: −46–20) [T(11) = 4.42, p < .05] and tended to be greater than in the ANA (95% CI: −80 – -5) [T(3) = 3.55, *p* = .076] (Fig. 4 upper center). We found no significant between-group differences during ankle movements [ALJ (15) vs. HLJ (95% CI: −35–7): T(11) = 0.71, p = .99; ALJ vs. ANA (95% CI: 21–105): T(3) = 0.49, *p* = 1.00] (Fig. 4 upper left) or hip movements [ALJ (17) vs. HLJ (95% CI: −21–115): T(11) = 0.23, p = 1.00; ALJ vs. ANA (95% CI: −50–45): T(3) = 0.18, p = 1.00] (Fig. 4 upper right). These results indicated that activated area in M1 ipsilateral to the movement side during knee movement when using the take-off/prosthetic leg was greater in the ALJ than other groups, but not during ankle and hip movements.

### 3.7 Activated area in the contralateral M1

We found no significant between-group differences during ankle movements [ALJ (−89) vs. HLJ (95% CI: −47–38): T(11) = 1.07, *p* = .61; ALJ vs. ANA (95% CI: 28–201): T(3) = 1.02, *p* = .77] (Fig. 4 lower left), knee movements [ALJ (154) vs. HLJ (95% CI: −13–82): T(11) = 1.32, *p* = .43; ALJ vs. ANA (95% CI: 23–163): T(3) = 0.40, p = 1.00] (Fig. 4 lower center), or hip movements [ALJ (−110) vs. HLJ (95% CI: −25–114): T(11) = 1.14, *p* = .56; ALJ vs. ANA (95% CI: 120–219): T(3) = 2.61, *p* = .16] (Fig. 4 lower right). These results indicated that the difference in the activated area in M1 contralateral to the movement side between the take-off/prosthetic and non-take-off/intact leg movements were comparable between groups even for knee movement.

### 3.8 Characteristics of representative participants

Representative participants are depicted in Fig. 2, in which we reported the values of each analysis. Differences in *t*-values for subject 7 of the HLJ (Fig. 3) were as follows: ipsilateral ankle: −2.29; ipsilateral knee: −1.06; ipsilateral hip: −0.41; contralateral ankle: −1.23; contralateral knee: −1.75; contralateral hip: −0.09. Differences in activated areas (Fig. 4) were as follows: ipsilateral ankle: −109; ipsilateral knee: −86; ipsilateral hip: 134; contralateral ankle: −74; contralateral knee: −100; contralateral hip: 73. Differences in *t*-values for subject 2 of the ANA (Fig. 3) were as follows: ipsilateral ankle: 0.12; ipsilateral knee: −1.03; ipsilateral hip: 0.59; contralateral ankle: 0.15; contralateral knee: −1.06; contralateral hip: 0.21. Differences in activated areas (Fig. 4) were as follows: ipsilateral ankle: −37; ipsilateral knee: −123; ipsilateral hip: 105; contralateral ankle: 100; contralateral knee: −73; contralateral hip: 186.

### 3.9 No correlation between brain activity and athletic performance

We tested the correlations between best record and 12 indices (i.e. ipsilateral and contralateral M1 × *t*-values and activated areas × ankle, knee and hip movements). We found no significant correlation between brain activity and the best record in the HLJ (all |r's| < 0.5, p's > 0.1).

### 3.10 Head movement during the task

The amount of volume-to-volume head movements did not differ between the six movement conditions (all p's > 0.37). The amount of volume-to-volume head movements in the ALJ was not significantly different to that of other groups in any six movement conditions. The maximum head movements were <2.5 mm in all scans in all participants [ALJ: x = 0.41 mm, y = 0.24 mm, z = 0.70 mm; HLJ: x = 0.24 ± 0.09 mm (range: 0.12–0.48 mm), y = 0.35 ± 0.16 mm (0.21–0.76 mm), z = 1.16 ± 0.57 mm (0.36–2.17 mm); ANA: x = 0.35 ± 0.19 mm (0.17–0.52 mm), y = 0.57 ± 0.26 mm (0.27–0.83 mm), z = 1.31 ± 0.49 mm (0.65–1.75 mm)]. The maximum head movements did not differ between the three groups (all p's > 0.32). There was no significant correlation between brain activity and head movements (all p's > 0.35).

### 3.11 Muscle activity during the task

Muscle activities irrelevant to unilateral muscle contraction did not differ between the three groups or between the tasks (all p's > 0.19) (Table 4).

**Table 4:** Muscle activities during the motor tasks.

	Soleus						Ractus femoris						Gluteus maximus					
	Take-off / prosthetic			Non take-off / intact			Take-off / posthetic			Non take-off / intact			Take-off / prosthetic			Non take-off / intact		
	ALJ	HLJ	ANA	ALJ	HLJ	ANA	ALJ	HLJ	ANA	ALJ	HLJ	ANA	ALJ	HLJ	ANA	ALJ	HLJ	ANA
T/P ankle		351 ± 104^⁎^		114	125 ± 54	96 ± 22	90	123 ± 55	95 ± 6	106	106 ± 15	88 ± 28	96	94 ± 25	89 ± 13	107	109 ± 31	90 ± 12
N/I ankle		154 ± 121		655	428 ± 246 _†_ ^∗^	341 ± 133	108	107 ± 18	104 ± 13 _†_ ^∗^	106	124 ± 32	110 ± 32	104	96 ± 27	102 ± 23	98	109 ± 28	112 ± 15
T/P knee		100 ± 24		105	92 ± 24	87 ± 28	150	334 ± 174	233 ± 79	108	95 ± 21	85 ± 31	111	114 ± 56	111 ± 37	102	103 ± 47	98 ± 38
N/I knee		102 ± 19		110	125 ± 57	122 ± 36	110	107 ± 23	101 ± 10	159	347 ± 159 _†_ ^∗^	244 ± 65	96	84 ± 25	99 ± 17	112	122 ± 70	111 ± 13
T/P hip		97 ± 21		100	97 ± 24	95 ± 16	106	131 ± 66	120 ± 45	108	94 ± 16	95 ± 12	622	573 ± 459^⁎^	628 ± 322	98	150 ± 124	131 ± 31
N/I hip		100 ± 22		102	100 ± 22	111 ± 28	108	120 ± 48	105 ± 26	124	144 ± 84	128 ± 53	96	110 ± 75	122 ± 41	586	534 ± 347*	385 ± 152

Values are expressed as a relative value (percentage) normalized to the value in the resting condition (mean ± one standard deviation). Asterisk symbols (*) represent a significant difference as compared with rest (100). Dagger symbols (†) represent a significant difference between the ALJ and HLJ. T/P: Take-off/Prosthetic side. N/I: Non-take-off/Intact side.

## 4 Discussion

We investigated brain activity during unilateral joint movements involving the lower extremities in a world's best Paralympic long jumper with a unilateral below-knee amputation. Twelve healthy long jumpers and four non-athletes with unilateral below-knee amputations performed the same tasks except for ankle movement. Our main finding was that activity in the ipsilateral M1 during knee movement of the take-off/prosthetic leg was greater in the ALJ than in the HLJ, and not during knee movement with the non-take-off/intact leg. In addition, in both ipsilateral activity and activated areas, differences between knee movement with the take-off/prosthetic and the non-take-off/intact legs were greater in the ALJ than in the HLJ or ANA. These results suggest that knee movements of the prosthetic leg in the ALJ had resulted in plastic changes in the ipsilateral M1. This functional plasticity of M1 ipsilateral to the take-off/prosthetic leg was likely caused by a combination of lower leg amputation below the knee and the intensive, long-term motor practice necessary to develop adequate control over the prosthesis and develop a superior long jump. No such differences were found in activity during ankle and hip movements between the ALJ and the other groups, which indicates that enhancement of activity for the knee movement with the take-off/prosthetic leg in the ipsilateral M1 was muscle specific. Previous studies in both macaque monkeys with spinal cord injury and older adult humans have suggested that activation in the ipsilateral M1 is a compensatory mechanism for impaired motor function (Nishimura et al., 2007; Zimerman et al., 2014). Our findings indicate that the ipsilateral M1 can be activated not only to compensate for impaired motor function but also to optimize the specific leg-prosthesis (coordinated) movements required for a high level of athletic performance.

Activity in the ipsilateral M1 was shown to be associated with controlling the timing of hand muscle recruitment (Davare et al., 2007). In addition, movement can be decoded with activity in the ipsilateral M1, which suggests that the ipsilateral M1 receives motor commands from higher motor regions that also input the contralateral M1 (Diedrichsen et al., 2013; Haar et al., 2015, Haar et al., 2017). Furthermore, ipsilateral pyramidal tract neurons have been found to assist contralateral pyramidal tract neurons (Jankowska et al., 2005). The ipsilateral M1 thus plays a role in motor control via the corpus callosum or ipsilateral tract (Jankowska et al., 2005; Waters et al., 2017). A recent electrophysiological study with rodents demonstrated that 3 months of skilled motor training with the right forelimb expanded the representation of the trained forelimb in the right (ipsilateral) motor cortex (Pruitt et al., 2016). A human study also found the similar plastic changes in the ipsilateral M1, which were evaluated with the amplitude of motor evoked potentials (Christiansen et al., 2017). These findings suggest that the recruitment of the ipsilateral M1 increases with long-term motor practice. In the present study, therefore, enhancement of activity and activated areas for knee movement with the take-off/prosthetic leg in the ipsilateral M1 might reflect the motor competence following training of knee movements with the prosthetic leg (i.e. prosthesis control and explosive power production). However, the contribution of the ipsilateral M1 to jump performance remains unclear. Indeed, a recent review underlined the fact that expansion of activation induced by amputation does not necessarily improve motor/sensory function (Makin and Bensmaia, 2017). Future longitudinal studies or those involving virtual lesion techniques will be needed to clarify this.

Our finding of use-dependent neural modulation is consistent with a previous study, which found that long-term, intensive use of the unilateral hand in violin players results in an expansion of the representation of the left fingers in the right motor and somatosensory cortices (Schwenkreis et al., 2007). This resulted in a significant right-left difference in these musicians. However, it should be noted that the direction of plasticity was opposite to that found in the present study; violin training induced a greater difference between the two hemispheres (i.e. lateralized), whereas long jump training in the present study reduced this (i.e. less lateralized). Although it is difficult to directly compare these two studies, the possible explanation for the difference might be associated with the type of motor training/task as well as with or without amputation. The ALJ has been trained to produce explosive power by using the prosthesis. In addition, the long jump take-off can be regarded as a unilateral movement (i.e. control of the non-take-off leg is not so important for take-off). On the other hand, the violinists must control their left fingers precisely and accurately as well as their right hands/arms simultaneously and independently. Future studies are needed to clarify why lower limb amputation and intensive, long-term motor practice induced plastic changes in M1 ipsilateral to the amputated leg rather than in the contralateral M1.

The task demand can affect activation of the ipsilateral M1 (Buetefisch et al., 2014; Wischnewski et al., 2016). However, the task demand should not be different between take-off and non-take-off leg movements (except for the ankle task). That is, it is unlikely that conducting knee muscle contraction with the take-off/amputated side was more demanding than doing so with the non-take-off/intact side because the knee joint and knee extensor muscles were intact in the amputated leg. Therefore, it could be that the greater difference in ipsilateral M1 activity between take-off and non-take-off leg movements in the ALJ was not due to the greater task demand or difficulty.

Brain activity in relevant motor areas of professional sports players and musicians is less widespread than that seen in individuals with lower motor skills, which indicates that they can move their limbs very efficiently (Krings et al., 2000; Naito and Hirose, 2014). However, we found that M1 activity during knee movements of the take-off/prosthetic leg was greater in the ALJ than the HLJ and ANA. This difference might be explained by differences in our motor tasks compared with previous ones. That is, neural efficacy and expansion of representation (or an increase in the total number of recruited neurons) progress simultaneously by motor learning and reflect different aspects (Karni et al., 1995; Pascual-Leone et al., 1995; Krings et al., 2000; Nakata et al., 2010; Dayan and Cohen, 2011; Naito and Hirose, 2014; Mizuguchi and Kanosue, 2017). Thus, we can predict that during a simple motor task brain activity would reflect the neural efficacy rather than the expansion of representation because the task can be performed using fewer neurons. However, for an unfamiliar motor task with high demands, the total number of neurons possible for recruitment would be activated. In the present study, participants were asked to perform the required movement with temporal accuracy as well as with the same contraction intensity on the right and left sides. In addition, the motor task (i.e. rhythmic isometric contraction of only one muscle group) was an unusual movement for all participants. These requirements mean that the current task was potentially more demanding than that of previous studies which required only an ankle rotation movement. Therefore, the participants' brains might have had to engage in more resources to perform these precise movements. Thus, a stronger and broader activation of the ipsilateral M1 in the ALJ could be interpreted as expansion of representation, rather than a weaker and more limited activation (i.e. neural efficacy).

Limb amputation induces dramatic cortical plasticity and reorganization. These plastic changes likely originate from unmasking inactive connections, axonal regeneration, and sprouting in the synapse architecture (Di Pino et al., 2009). A previous study using transcranial magnetic stimulation demonstrated that the cortical map for an amputated leg in the ipsilateral M1 was modulated after amputation of the lower limb (Schwenkreis et al., 2003). This suggests that limb amputation not only induces plasticity in the contralateral M1, but also in the ipsilateral M1. Plasticity in the ipsilateral M1 is also likely associated with an altered microstructure in the corpus callosum or subcortical regions (Makin and Bensmaia, 2017; Li et al., 2017). These findings support the possibility that limb amputation induces different activity in the ipsilateral M1 for prosthetic and intact limb movements. We found that differences in ipsilateral M1 activity and activated areas during knee movement were greater in the ALJ than the ANA. This suggests that intensive long-term motor practice after limb amputation of distal parts boosts neural plasticity in M1 ipsilateral to the amputated leg. In the present study, the ANA had been amputees for longer and had used a prosthetic leg for longer than the ALJ. If years of amputee was an important factor for enhancement of ipsilateral activity, we should find greater ipsilateral activity in the ANA rather than the ALJ. Therefore, a longer time spent as an amputee, and without intensive practice, is not sufficient to induce the drastic plasticity seen in M1 ipsilateral to the amputated leg. However, patients with a limb amputation would have a substantial potential for modifying in M1 ipsilateral to the amputated leg. Indeed, the ALJ started long jump training 5 years after amputation. This suggests that the ipsilateral M1 retains its capacity for functional plasticity even after several years have passed since limb amputation. These results indicate that, following amputation, extensive practice during motor rehabilitation that involves the use of the prosthesis is very important to induce cortical plasticity and thus optimize recovery of the lost motor functions. Unfortunately, the use of fMRI in the present study did not allow us to determine the detailed mechanisms underlying the enhancement of ipsilateral M1 activity. Furthermore, it remains unclear whether reorganization in the ipsilateral M1 actually occurred in the ALJ. To understand this, future work could determine whether the enhancement of ipsilateral M1 activity was induced by the contralateral M1 via the corpus callosum or by higher motor regions such as the supplementary motor area and/or premotor cortex or by subcortical regions as well as to evaluate the cortical representation of each body part.

M1 activity during motor imagery has been reported to be smaller than that during actual movements (Hétu et al., 2013). Therefore, it is difficult to compare brain activity during ankle movements between the ALJ and the HLJ. In addition, motor imagery of the amputated limb with phantom sensation activates a distinct network of brain areas compared to execution of the amputated/phantom limb (Raffin et al., 2012a, Raffin et al., 2012b). For example, M1 activity during movement execution of the amputated/phantom limb was greater than that during motor imagery (Raffin et al., 2012b). Therefore, execution of the amputated/phantom limb might be a better task than motor imagery to compare brain activity between amputated and intact limb movements. However, in patients with quadriplegia, M1 was clearly activated during motor imagery (Di Rienzo et al., 2014). The authors postulated that this occurred because these patients did not need to have an inhibition of M1 from higher motor regions to suppress muscle activity during motor imagery. This would explain why we found clear activation of M1 during motor imagery of ankle movement of the amputated leg, although other researchers have failed to find clear M1 activity during motor imagery in non-amputees (Hétu et al., 2013).

The whole-brain analysis also revealed that the ALJ exhibited greater activity in the thalamus and cerebellum than the HLJ. Previous studies have reported that limb amputation modulated activity not only in the cerebral cortex but also in subcortical regions, including the thalamus (Di Pino et al., 2009; Florence et al., 2000; Makin and Bensmaia, 2017). In addition, gray matter of the thalamus contralateral to the side of the amputation has been reported to decrease after limb amputation (Draganski et al., 2006), which indicates that amputation induced functional plasticity in ipsilateral subcortical regions as well as in the ipsilateral M1.

One limitation of the present study is that the number of participants was small. Although neural data from a Paralympic gold-medalist long jumper with a below-knee amputation are rare and valuable, the data are nonetheless limited, and future work could recruit more participants to verify the present findings with appropriate statistical power and correction for multiple comparison. Indeed, we performed Crawford-Howell *t*-tests 24 times [2 (ALJ vs. HLJ and ALJ vs. ANA) × 2 (individual and leg ROI) × 2 (contralateral and ipsilateral) × 3 (limbs)] per analysis. Thus, the significance threshold in the present study was relatively liberal. In the fMRI preprocessing, we did not use structural T1-weighted images because time constraints meant that we were not able to measure T1 images from all participants. Therefore, the accuracy of data registration was not optimal. In addition, the spatial resolution of scanning, non-linear transformation from native to MNI space, and ROI settings made it difficult to reliably distinguish between the M1 and primary somatosensory cortex (S1). Therefore, our results might have been influenced by S1 activity. A study using ultra-high resolution fMRI will be needed to clarify this issue. In the whole-brain analysis, we used a two-sample *t*-test rather than a Crawford-Howell *t*-test because SPM12 does not support the Crawford-Howell *t*-test. Therefore, careful interpretation is needed, even when the *p*-value of M1/S1 was 0.015 in the whole-brain analysis (Table 3). Another limitation is that the ALJ's performance level was superior to that of the HLJ. Therefore, it is possible that greater activity in M1/S1 ipsilateral to the take-off leg would be observed in world-class long jumpers; however, we found no correlation between performance level and brain activity during movements in the HLJ. To address this limitation, future work should recruit world-class long jumpers. Another limitation of the present work is that we did not record muscle activity during the MRI scan. While electromyographic (EMG) activity was recorded during movement practice, the participants were able to contract only the agonist muscle on one side before the MRI scan. Therefore, muscle activity during the MRI scan might have been different to that before the scan. However, we believe that activity level in the homologous muscles would be comparable because participants were instructed to contract their muscles with the same effort level. Indeed, we did not find greater difference in activity during the ankle and hip tasks in the ALJ. Therefore, the greater difference seen in knee movements of the ALJ is likely to reflect the increase in the number of neurons recruited for knee movement with the prosthetic take-off leg.

In summary, we found that activity in the ipsilateral M1/S1 associated with knee movements of the prosthetic leg was greater in the ALJ with below-knee amputation than in healthy long jumpers or non-athletes with below-knee amputation. This suggests that a combination of below-knee amputation and intensive long-term long jump training using a prosthesis (i.e. fine knee joint control as well as explosive power production) increased activity in the ipsilateral M1/S1 for take-off involving the prosthetic limb. These results provide novel insights into the potential for substantial cortical plasticity with an extensive motor training and use of prosthesis. In addition, the ALJ started long jump training 5 years after amputation, we can also conclude that functional plasticity in the ipsilateral M1/S1 occurs even after time has passed since limb amputation. These findings may inform the development of novel motor rehabilitation that better tap into the potential of cortical plasticity in amputees with a prosthesis.

## Author contributions

NM, KeN, KK, and KiN designed the research; KeN and YT performed experiment; NM and KeN analyzed the data; NM, KeN, YT, KK, and KiN wrote the paper.

## Declarations of interest

None.
